# Supplementary material for: Synthesis, In Silico, and Biological Evaluation of a Borinic Tryptophan-Derivative That Induces Melatonin-like Amelioration of Cognitive Deficit in Male Rat
Source: Int J Mol Sci. 2022 Mar 17;23(6):3229. doi: 10.3390/ijms23063229 (PMC8952423; doi:10.3390/ijms23063229)
Supplement: Supplementary file 1 [file ijms-23-03229-s001.zip › ijms-1595085-supplementary.pdf]

Supplementary material

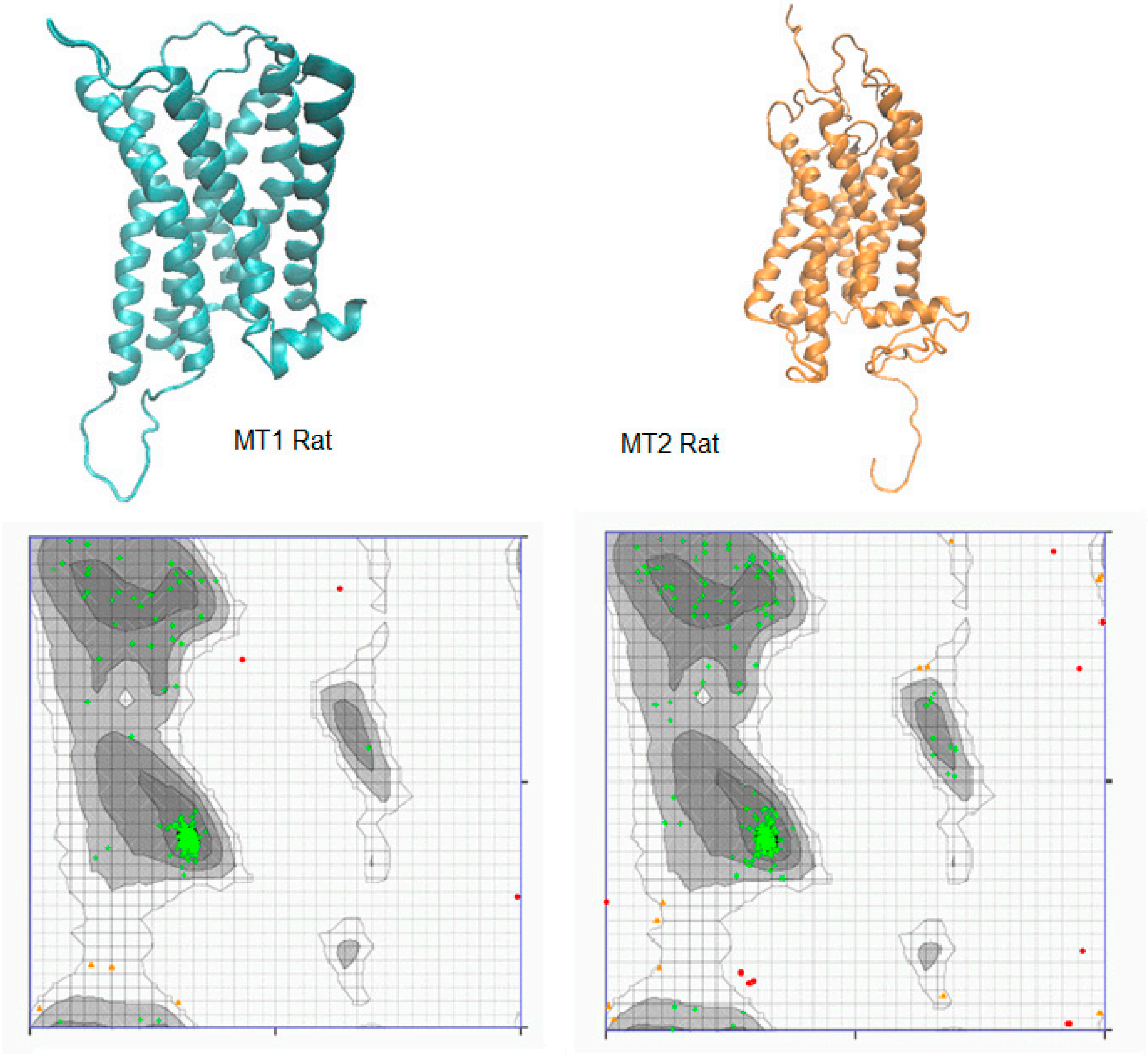

Figure S1. Ramachandran plots for built rat MT1 and MT2 receptors.

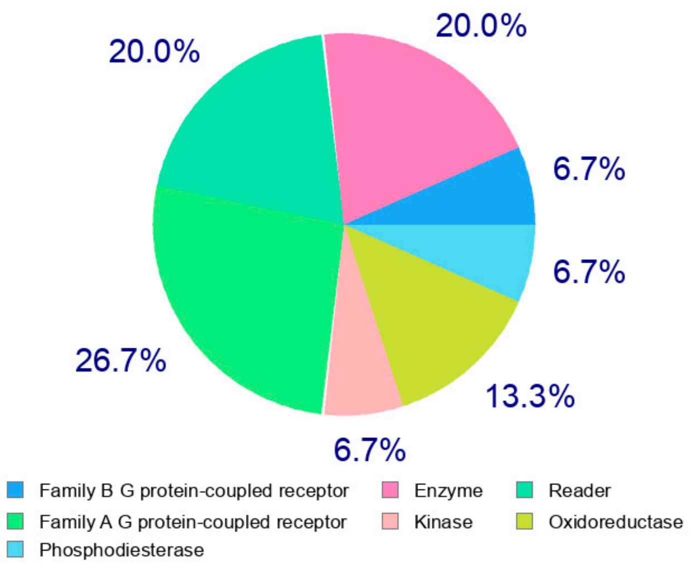

Figure S2. The Swiss-target prediction results regarding targets for boromelatonin compound. The mentioned server predicted G-protein coupled receptors in family A as the group with higher probability of interaction for borolatonin. Results include melatonin, serotonin, histamine, purinergic and trace amine-associated receptors.

Table S1. Predicted and reported experimental affinity of well-known melatonin receptor ligands.

	On MT1 melatonin receptor		On MT2 melatonin receptor		
**Ligand**	Theoretical	Experimental [a]	Ligand	Theoretical	Experimental [a]
LUZINDOLE	3.84	6.5	S26131	4.08	6.9
S20928	3.93	6.65	5-HEAT	5.71	7.1
-PDOT	3.96	6.7	S20928	4.23	7.15
AZD7325	4.08	6.9	LUZINDOLE	4.65	7.85
UCM724	4.08	6.9	UCM724	4.74	8
K185	4.26	7.2	S22153	4.83	8.15
5-HEAT	5.35	7.8	HYDROXYMELATONIN	5.64	8.5
UCM549	4.68	7.9	UCM549	5.36	9.05
S22153	4.71	7.95	4P-PDOT	5.39	9.1
CHLOROMELATONIN	5.20	8	UCM793	5.20	9.2
S26131	5.24	8.85	K185	5.51	9.3
HYDROXYMELATONIN	5.27	9.1	EFPPEA	6.52	9.4
UCM793	4.98	9.1	CHLOROMELATONIN	5.42	9.6
MELATONIN	5.06	9.4	MELATONIN	5.09	9.6
TASIMELTEON	6.01	9.5	GR196429	6.15	9.75
GR196429	5.79	9.7	IODOMELATONINE	6.01	10
TIK-301	5.64	10.1	RAMELTEON	5.84	10
EFPPEA	5.86	10.2	TASIMELTEON	6.30	10.2
IODOMELATONINE	5.57	10.2	AGOMELATINE	6.31	10.2
AGOMELATINE	5.88	10.2	TIK-301	5.79	10.4
METHOXY-DIDEHYDRO-AGOMELATINE	6.15	10.5			
RAMELTEON	5.56	10.9			

[a] Experimental values were taken from IUPHAR-database [32].
